# Incidence of Hansen’s Disease — United States, 1994–2011

**Published:** 2014-10-31

**Authors:** Leisha Nolen, Dana Haberling, David Scollard, Richard Truman, Alfonso Rodriguez-Lainz, Laura Blum, David Blaney

**Affiliations:** 1 Division of High-Consequence Pathogens and Pathology, National Center for Emerging and Zoonotic Infectious Diseases, CDC; 2 National Hansen’s Disease Programs, Health Resources and Services Administration; 3 Division of Global Migration and Quarantine, CDC; 4 University of California, Berkeley

Hansen’s disease (HD), or leprosy, is caused by the bacterium *Mycobacterium leprae* and is reportable in many states. It is a chronic disease affecting the skin and nerves, commonly presenting as pale or reddish skin patches with diminished sensation. Without treatment, it can progress to a severely debilitating disease with nerve damage, tissue destruction, and functional loss. An important factor in limiting HD morbidity is early diagnosis and prompt initiation of therapy. Because HD is rare, clinicians in the United States are often unfamiliar with it; however, HD continues to cause morbidity in the United States. To better characterize at-risk U.S. populations, HD trends during 1994–2011 were evaluated by reviewing records from the National Hansen’s Disease Program (NHDP). When the periods 1994–1996 and 2009–2011 were compared, a decline in the rate for new diagnoses from 0.52 to 0.43 per million was observed. The rate among foreign-born persons decreased from 3.66 to 2.29, whereas the rate among U.S.-born persons was 0.16 in both 1994–1996 and 2009–2011. Delayed diagnosis was more common among foreign-born persons. Clinicians throughout the United States should familiarize themselves with the signs and symptoms of HD and understand that HD can occur in the United States.

Although not highly contagious, HD is thought to be transmitted through nasal secretions (1). The normal incubation period ranges from 3 to 7 years (2). Initial presentation is often one or more chronic anesthetic macular or maculopapular skin lesions. HD can progress to involve peripheral nerves, resulting in sensory and motor loss, and ultimately to permanent disability (3).* Since 1991, the World Health Organization has led a global campaign to eliminate HD as a public health problem, with elimination defined as a worldwide prevalence of <10 cases per 100,000 persons (4).

For this report, the NHDP registry of new HD diagnoses during 1994–2011 was examined. The population was divided into U.S.-born and foreign-born persons. Persons born within the United States and its territories were considered U.S.-born, and those born outside of the United States and its territories were considered foreign-born. Cases among persons listed as living outside of the United States and persons missing the date of diagnosis or the place of birth were excluded from the review; cases missing date of symptom onset or date of entry to the United States were excluded from relevant analyses.

The rates of new diagnoses of HD were calculated by country of birth for each year from the period 1994–2011. Poisson regression was used to compare rates between groups with rate ratios and 95% confidence intervals (CIs) and to analyze rate trends over time. All rates were calculated using population estimates obtained from the U.S. Census Bureau’s Current Population Survey (5). The annual rate of new diagnosis of HD in persons in the United States from specific regions of the world was analyzed for the period 2007–2011 using population estimates from the U.S. Census Bureau American Community Survey.

The number of years between onset of symptoms and diagnosis (i.e., the delay), was calculated. The exact reported dates of onset were available, but were found to be significantly biased to January 1 as the onset day and month. This might reflect the ability of patients to identify the year their symptoms started but not the month or day. For this reason, data were analyzed by year alone.

During 1994–2011, there were 2,323 new cases of HD, with an average annual incidence rate of 0.45 cases per 1 million persons (CI = 0.43–0.47). A 17% decrease in the rate of new diagnoses was observed for the U.S. population overall, from 0.52 (CI = 0.47–0.57) during 1994–1996 to 0.43 (CI = 0.39–0.48) during 2009–2011 (Figure 1). During 1994–2011, U.S.-born persons had an average annual rate of 0.13 (CI = 0.12–0.14), but the rate for 1994–1996 was 0.16 (CI = 0.13–0.19) and was the same for 2009–2011 (0.16 [CI = 0.14–0.19]). Foreign-born persons had an average rate of 2.81 (CI = 2.67–2.95) during 1994–2011 but a higher rate (3.66 [CI = 3.23 – 4.15]) for 1994–1996 and a lower rate (2.29 [CI = 2.02–2.58]) for 2009–2011.

The U.S. census began including country of birth in its data in 2007; therefore, the analysis regarding country of birth was limited to 2007–2011. During this 5-year period there were 677 new diagnoses of HD in the United States, of which 461 (68%) included data regarding country of birth (Table). Persons born in Oceania had the highest rate of HD diagnosis during this period, with an average annual rate of 556.9 cases per 1 million population, more than 10 times the rate observed for any other region. Ninety-seven percent of those diagnosed from Oceania were born in the Federated States of Micronesia or the Marshall Islands, and almost half of these persons (48.9%) were diagnosed in Hawaii.

The number of years from onset of symptoms to HD diagnosis was calculated for 2,124 cases. Most (74%) patients had a delay of <3 years between symptom onset and diagnosis; however, some were not diagnosed for many years after the onset of symptoms (Figure 2). The median delay was 1 year, and the mean delay was 2.4 years. Among foreign-born persons, 232 (18%) reported onset of symptoms before their entry into the United States, with a median delay of 2.5 years (mean = 5.7 years). These data differed significantly from the median delay for U.S.-born persons and foreign-born persons who had the onset of symptoms at or after entry, both of whom had a median of 1 year (p<0.001).

## Discussion

HD continues to occur in the United States, both in U.S.-born and foreign-born persons. Many clinicians are not familiar with this disease or its manifestations and treatments, but *M. leprae* is an important pathogen to consider when caring for patients with chronic skin disorders of unknown cause. The rate of diagnosis of HD among foreign-born persons during 2009–2011 was 14 times higher than among U.S.-born persons. From 1994–1996 to 2009–2011, the rate of diagnosis in foreign-born persons decreased by 37%. Although 70% of HD diagnoses occur in persons born outside of the United States, *M. leprae* continues to cause disease in U.S.-born persons, with an average of 56 cases diagnosed in U.S.-born persons in the United States each year during 2009–2011.

Persons born in Oceania were identified as the population with the highest rate of HD within the United States, with a rate of diagnosis >10 times that of any other region, in agreement with previous reports (6). This highlights the need for investment in HD-related training, resources, and surveillance in Oceania. Hawaii was identified as the location where most of the patients from Oceania were diagnosed; however, clinicians throughout the United States should be encouraged to consider HD when evaluating chronic skin conditions, especially in patients from this region.

HD is one of the diseases for which prospective immigrants to the United States are screened.† However, persons who come to the United States but do not apply for permanent residency or come without authorization do not undergo this screening. In addition, only 18% of foreign-born persons diagnosed with HD in the United States reported having symptoms before their admission into the United States, making it unlikely that many cases will be detected by immigrant screening. Therefore, primary care clinicians need to be aware of HD and its manifestations to adequately monitor recent immigrants.

HD can lead to severe nerve and tissue damage if treatment is delayed for months or years; for this reason it is important to recognize this disease and begin treatment as early as possible. While most patients were diagnosed within 1 year of the onset of symptoms, many patients had symptoms for many years before diagnosis. Foreign-born persons were at the highest risk for delayed diagnosis.

The findings in this report are subject to at least two limitations. First, data are limited to those patients reported to NHDP.§ This program not only collects information from outside providers regarding HD diagnoses, but also provides free medications to HD patients within the United States. As a result, it is expected that most patients with HD in the United States are reported to NHDP. Second, data regarding the onset of symptoms are limited by patient recall; many patients report the onset of symptoms many years before contact with NHDP. It is possible that some of the symptoms they attribute to HD might have been caused by other diseases or problems.

To decrease HD in the United States, diagnosis and treatment needs to be improved among persons in the United States who were born in countries where there is a high prevalence of HD and also in those countries themselves. Clinicians throughout the United States should be aware of the signs and symptoms of HD and know that HD exists in the United States. It is important to consider this disease when evaluating chronic skin conditions, especially those with associated loss of sensation. By diagnosing and treating patients early, it is possible to prevent further transmission and lifelong disability.

What is already known on this topic?

Hansen’s disease (HD), or leprosy, is a reportable disease that can cause significant disability if not diagnosed and treated. During 1994–1996, the annual incidence of newly diagnosed HD in the United States was 0.52 cases per 1 million population. Clinicians in the United States are often unfamiliar with HD, resulting in delayed diagnosis and treatment.

What is added by this report?

During 2009–2011, the annual incidence of newly diagnosed HD in the United States was 0.43 cases per 1 million population. Foreign-born persons living in the United States had a rate of HD diagnosis 14 times higher than U.S.-born persons, with those born in Oceania having the highest rate of diagnosis. One fourth of HD patients had symptoms for >3 years before they were diagnosed; delayed diagnoses were more common among foreign-born persons. This report helps inform clinicians of the signs and symptoms to be aware of when evaluating at-risk patients for HD.

What are the implications for public health practice?

HD is an important disease to consider when treating anyone in the United States with a chronic skin condition. Clinicians who routinely treat patients who are foreign-born, especially those from Oceania, should keep HD in mind. Detecting and treating HD in countries where it is most common is one strategy for reducing the incidence of HD in the United States.

## Figures and Tables

**FIGURE 1:**
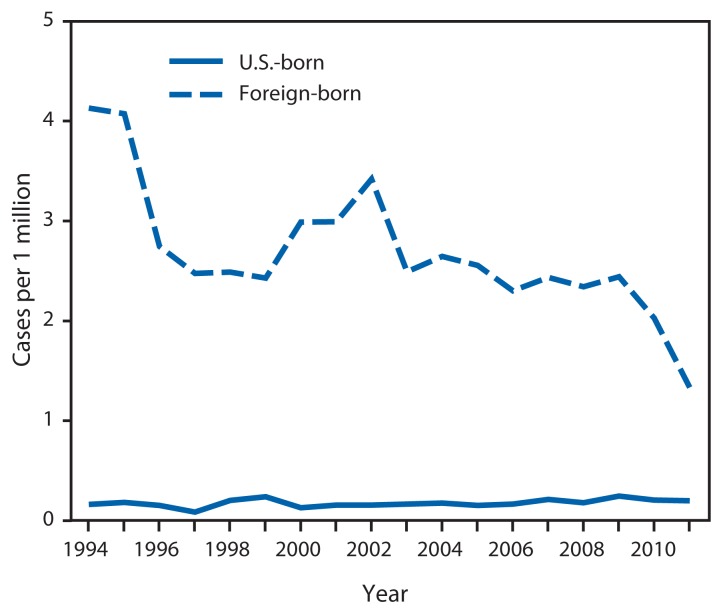
Rate of new diagnoses of Hansen’s disease, by U.S. birth status — United States, 1994–2011

**FIGURE 2:**
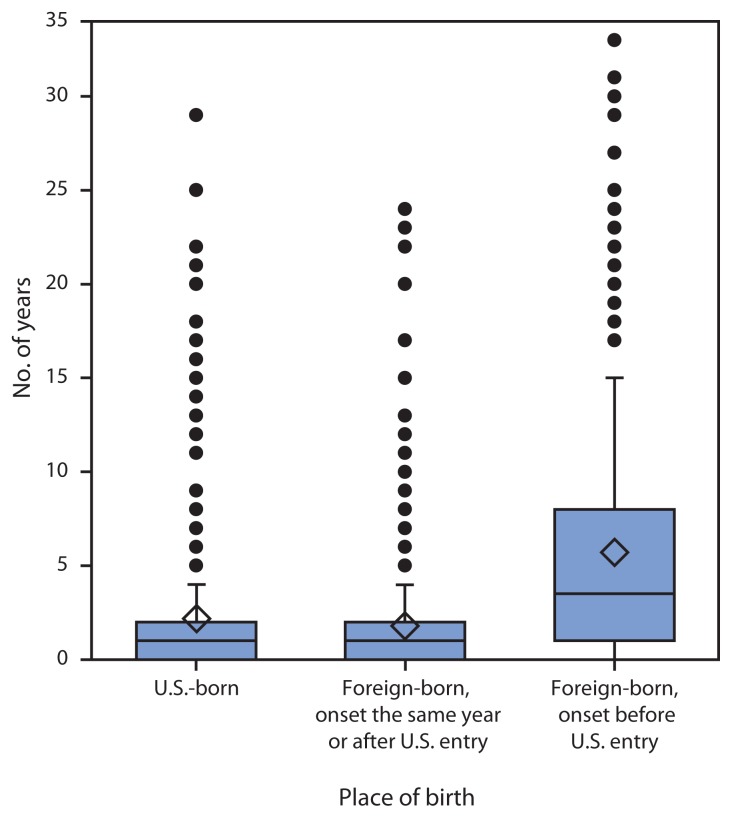
Period from year of symptom onset to Hansen’s disease diagnosis, by place of birth — United States, 1994–2011* * The horizontal line inside each box represents the median, the diamond indicates the mean number of years, and the top and bottom of the box are the first and third quartile. The whiskers are extended to 1.5 and 3.5 quartiles. The dots represent individual outliers. The mean period from symptom onset to diagnosis for foreign-born persons with onset before entering the United States is statistically different from the mean period for foreign-born persons with onset the same year or after entry and the mean period for U.S.-born persons (p<0.001).

**TABLE:** Number and rate* of new Hansen’s disease diagnoses among foreign-born persons, by region of birth — United States, 2007–2011

Region of birth	No. of new diagnoses	Rate	(95% CI)
Europe	1	0.21†	(0.011–1.34)
North America	109	5.74	(4.74–6.96)
Asia	117	10.61	(8.81–12.76)
Africa	22	14.47	(9.30–22.30)
South America	89	33.34	(26.93–41.23)
Oceania	123	556.93	(464.78–666.86)

**Abbreviation:** CI = confidence interval.

* Rate per 1 million population.

† Rate estimate is limited because there was only one new diagnosis in the European-born population.

## Footnotes

* Additional information available at http://www.cdc.gov/leprosy.

† Additional information available at http://www.cdc.gov/immigrantrefugeehealth/exams/ti/panel/technical-instructions/panel-physicians/hansens-disease.html.

§ Additional information available at http://www.hrsa.gov/hansensdisease.
